# Oxidative Stress-Protective and Anti-Melanogenic Effects of Loliolide and Ethanol Extract from Fresh Water Green Algae, *Prasiola japonica*

**DOI:** 10.3390/ijms19092825

**Published:** 2018-09-18

**Authors:** Sang Hee Park, Eunju Choi, Sunggyu Kim, Dong Sam Kim, Ji Hyeon Kim, SeokGu Chang, Jae Seok Choi, Kyung Ja Park, Kyung-Baeg Roh, Jongsung Lee, Byong Chul Yoo, Jae Youl Cho

**Affiliations:** 1 Department of Biocosmetics, Sungkyunkwan University, Suwon 16419, Korea; 84701@naver.com (S.H.P.); sukim590@skku.edu (S.K.); 2 Department of Integrative Biotechnology, Sungkyunkwan University, Suwon 16419, Korea; cej223@naver.com; 3 Research and Business Foundation, Sungkyunkwan University, Suwon 16419, Korea; 4 Samcheok Prasiola Japonica Research Center, Samcheok City Hall, Samcheok 25914, Korea; prasiolra@korea.kr (D.S.K.); kjh0512@korea.kr (J.H.K.); jangsg69@korea.kr (S.C.); kyu5132@korea.kr (K.J.P.); 5 Environmental Research Institute, Kangwon National University, Chuncheon 24341, Korea; gobiobotia@kangwon.ac.kr; 6 Biospectrum Life Science Institute, Yongin 1682, Korea; biosh@biospectrum.com; 7 Biomarker Branch, Research Institute, National Cancer Center, Goyang 10408, Korea; 8 Department of Cancer Biomedical Science, Graduate School of Cancer Science and Policy, National Cancer Center, Goyang 10408, Korea

**Keywords:** loliolide, *Prasiola japonica*, antioxidant, anti-melanogenesis

## Abstract

Loliolide is a monoterpenoid hydroxylactone found in many algae, including fresh water green algae, *Prasiola japonica*. To date, loliolide and compounds in *P. japonica* have not been studied systematically with respect to skin pharmacology. In this study, we investigated oxidative stress-protective and anti-melanogenic effects of loliolide and *P. japonica* ethanol extract (Pj-EE), known to contain loliolide, in human keratinocyte (HaCaT) cells and mouse melanoma (B16F10) cells. Loliolide suppressed the transcription of genes encoding matrix metalloproteinases (MMPS), which were induced in HaCaT cells by hydrogen peroxide (H_2_O_2_) treatment. Loliolide and Pj-EE not only reduced the melanin secretion and content in B16F10 cells but also increased the expression of the antioxidant proteins nuclear factor (erythroid-derived 2)-like 2 (NRF2) and heme oxygenase-1 (HO-1) in HaCaT cells subjected to H_2_O_2_ treatment. Furthermore, loliolide and Pj-EE decreased expression of the anti-melanogenic protein microphthalmia-associated transcription factor (MITF) and tyrosinase in B16F10 cells subjected to α-melanocyte-stimulating hormone (α-MSH) treatment. Our findings demonstrate that loliolide and Pj-EE have antioxidant and anti-melanogenic effects on skin.

## 1. Introduction

Skin is an important organ that protects the human body from external factors and damage [1]. Reactive oxygen species (ROS), which induce oxidative stress, are important molecules involved in skin aging. ROS has been reported as a key molecule that causes skin cell death, wrinkling, and pigmentation [2,3,4,5]. Together with ROS, upregulated expression of matrix metalloproteinases (MMPs) causes wrinkle formation; elevated expression of cyclooxygenase (COX)-2, leading to an inflammatory reaction; and increased secretion of α-melanocyte-stimulating hormone (α-MSH) in keratinocytes, leading to melanogenic responses [6].

Since serious damage results when cells are exposed to oxidative stress, cellular antioxidants are employed as molecular defense responses. Increased expression of hemeoxygenase 1 (HO-1), which is activated by the nuclear factor (erythroid-derived 2)-like 2 (NRF2)-Kelch-like ECH-associated protein 1 (KEAP1) signaling pathway, is also a defensive response [7]. The NRF2-KEAP1 signaling pathway is activated by a signaling cascade composed of phosphatidylinositol-4,5-bisphosphate 3-kinase (PI3K) and protein kinase B (AKT). In normal cells, NRF2-KEAP1 is inactivated by the interaction of the two proteins, but, after exposure to oxidative stress, the NRF2-KEAP1 interaction is abolished, and the separated NRF2 binds to the antioxidant response elements (AREs) of the *HO-1* promoter to activate its expression. Expression of *HO-1* produces bilirubin working as an endogenous antioxidant molecule [8]. Tyrosinase protein has been the focus of research on melanogenesis. Tyrosinase is produced by the melanocortin 1 receptor (MC1R) signaling pathway and plays a central role in melanin production. In normal melanocytes, the MC1R signaling pathway is activated by α-MSH. After MC1R activation, a series of intracellular signaling pathway composed of adenylyl cylase, its product, cAMP, and protein kinase A (PKA) participate in the activation of cAMP response element-binding (CREB) protein, and then microphthalmia-associated transcription factor (MITF) are sequentially expressed by CREB. Finally, synthesized MITF controls the expression of various genes such as tyrosinase, tyrosinase-related protein-1 (TRP)-1, and TRP-2 that are essential for melanin synthesis and secretion in melanocytes [9,10,11,12].

**Figure 1:**
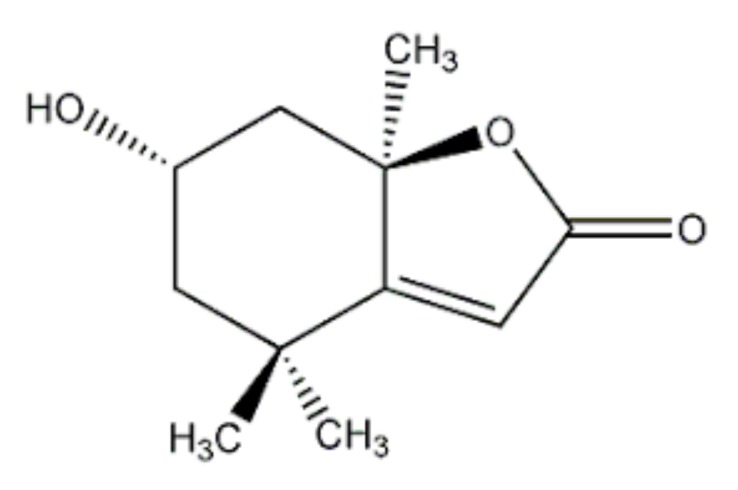
Structure of loliolide.

Green algae is considered as one of representative biosources to be applied for preparation of pharmaceutical, neutraceutical, and cosmoceutical products [13,14]. *Prasiola japonica* is fresh water green algae with a lot of compounds such as (−)-loliolide (Figure 1), methyl pyrazine, 1-hydroxy-2-propanone, diisopropylamine, 1,6-dihydro-6-oxo-3-pyridinecarboxamide, mannitol, mannose, and glucitol, according to gas chromatography/Mass spectrometric analysis [15]. Of them, loliolide is known to be an active component in green algae with various biological roles such as anti-aging, anti-viral, and anti-inflammatory activities [16,17,18,19]. So far, however, the effects of fresh water green algae components, loliolide and *P. japonica* ethanol extract (Pj-EE), on the skin have not been studied extensively [20]. In this study, we investigated oxidative stress-protective effects of loliolide and Pj-EE in human keratinocyte HaCaT cells. To do this, the effects of loliolide and Pj-EE on the expression of NRF2-KEAP1 signaling pathway proteins were confirmed by Western blot analysis. The expression of genes encoding MMPs was confirmed by reverse transcription-polymerase chain reaction (RT-PCR) and real-time PCR analyses, and cell viability was determined by 3-(4,5-dimethylthiazol-2-yl)-2,5-diphenyltetrazolium bromide (MTT) assay [21,22]. Additionally, mouse melanoma B16F10 cells were used to assess the anti-melanogenic effects of loliolide and Pj-EE by measuring level of melanin content and secretion [23,24]. The expression of MC1R proteins related to melanogenesis was also confirmed by Western blot analysis [25].

## 2. Results

### 2.1. Antioxidant Effect of Loliolide

**Figure 2:**
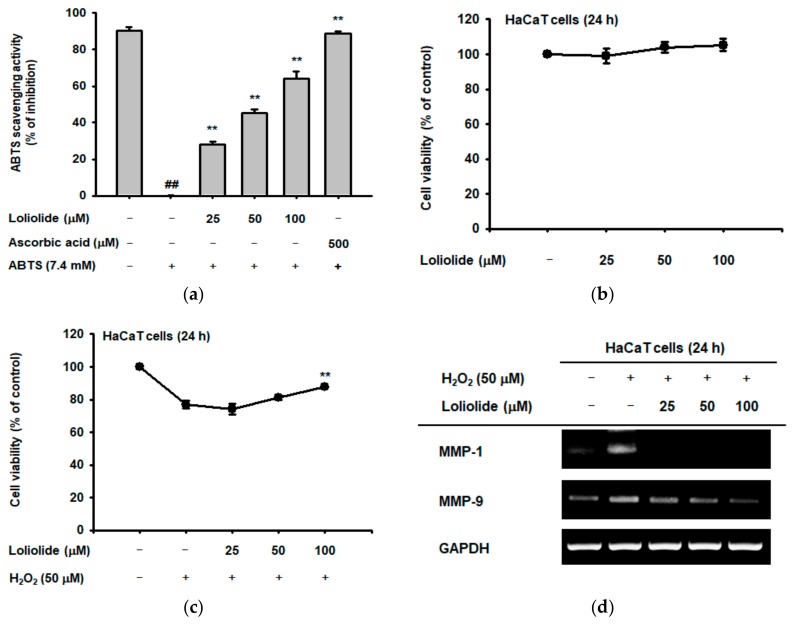

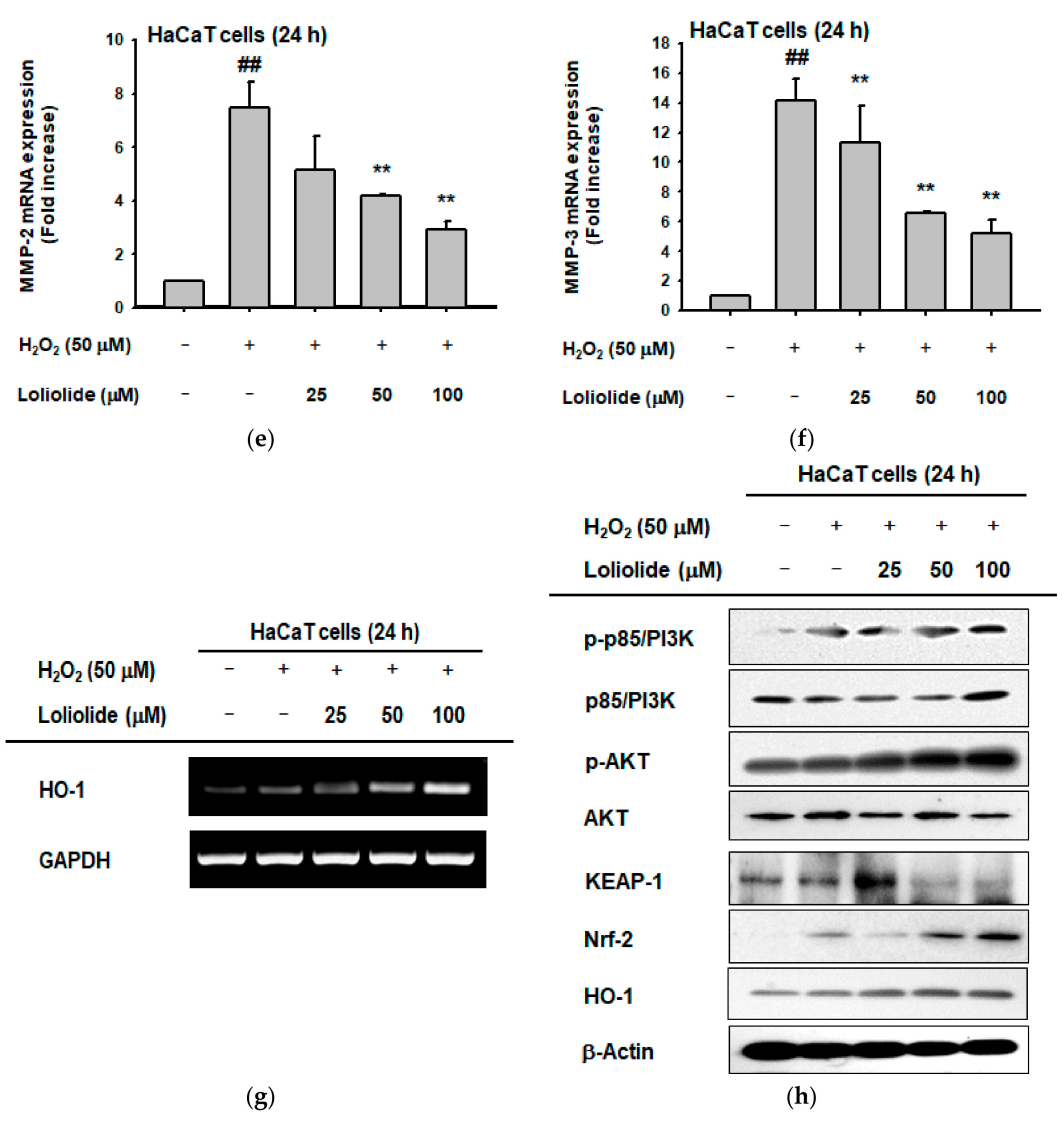
Oxidative stress-protective effect of loliolide on HaCaT cells. (**a**) Measurement of the ABTS radical scavenging activity of loliolide. (**b**,**c**) Viability of HaCaT cells treated with loliolide alone or loliolide plus H_2_O_2_ as measured by MTT assay. (**d**–**f**) RT-PCR and real-time PCR analyses of *MMP* gene expression in HaCaT cells treated with loliolide and H_2_O_2_. (**g**) RT-PCR analysis of *HO-1* gene expression in HaCaT cells treated with H_2_O_2_ and loliolide. (**h**) Western blot analysis of the expression of proteins in the NRF2-KEAP1 signaling pathway in H_2_O_2_- and/or loliolide-treated HaCaT cells. Statistical significance (**a**,**c**,**e**,**f**) was evaluated using the Kruskal–Wallis/Mann–Whitney test. ^##^ *p* < 0.05 compared with normal group and ** *p* < 0.01 compared with control.

We evaluated the antioxidant effects of loliolide using a 2,2′-azino-bis (3-ethylbenzothiazoline-6-sulphonic acid) (ABTS) assay, which can be useful to measure the level of hydroxyl radicals, with ascorbic acid as a positive control compound, since we have chosen H_2_O_2_ as an oxidative stress inducer [26]. Loliolide reduced the ABTS radical with an IC_50_ value of 61.52 ± 2.12 μM in the ABTS assay (Figure 2a) [27]. We determined the viability of HaCaT cells in the presence of loliolide using an MTT assay and found that cell viability was unaffected, even up to a concentration of 100 μM (Figure 2b). We also assessed the viability of HaCaT cells treated with loliolide and H_2_O_2_. Loliolide protected HaCaT cells against cell death caused by H_2_O_2_-induced oxidative stress in a dose-dependent manner (Figure 2c). Next, we examined the expression of genes encoding MMPs in HaCaT cells. MMPs are involved in senescence and cell death resulting from increased oxidative stress caused by ROS [28]. The expression of *MMPs* in HaCaT cells after treatment with H_2_O_2_ and loliolide was determined by RT-PCR and real-time PCR analyses. H_2_O_2_ treatment upregulated the expression of *MMPs*, but loliolide reduced the increase in *MMP* gene expression in a dose-dependent manner (Figure 2d–f). To confirm the antioxidative mechanism of loliolide, we treated HaCaT cells with H_2_O_2_ to induce oxidative stress. When the H_2_O_2_-treated cells were simultaneously treated with loliolide, the expression of *HO-1*, an important antioxidant gene [29], was increased in a dose-dependent manner (Figure 2g). We also examined the expression of proteins in the NRF2-KEAP1 signaling pathway in relationship to *HO-1* gene regulation. The expression levels of the PI3K, AKT, and NRF2 proteins in the NRF2-KEAP1 signaling pathway, and HO-1 were increased in HaCaT cells treated with H_2_O_2_ and/or loliolide (Figure 2h) [8].

### 2.2. Anti-Melanogenic Effect of Loliolide

**Figure 3:**
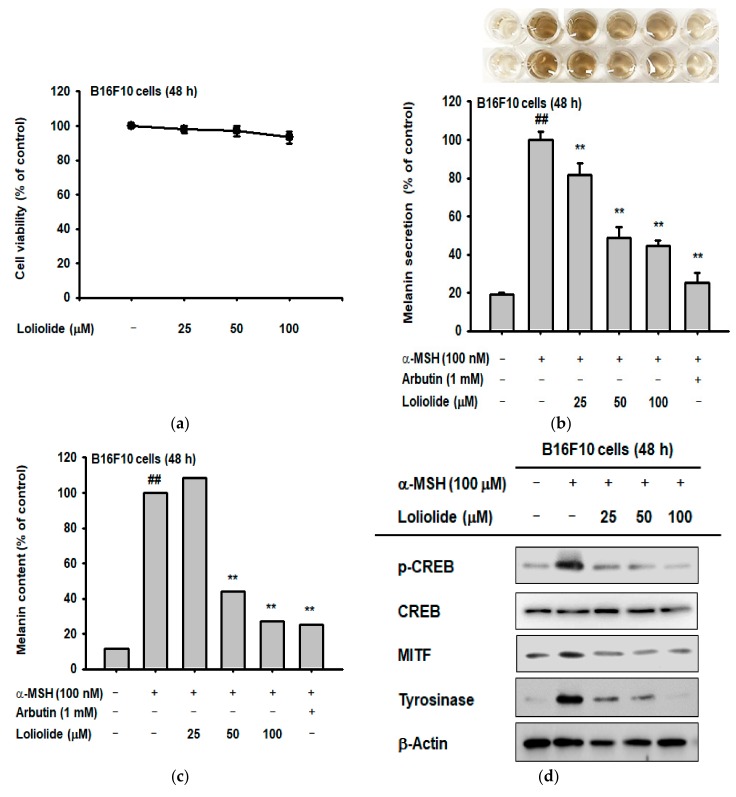
Anti-melanogenic effect of loliolide. (**a**) Measurement of cell viability by MTT assay in loliolide-treated B16F10 cells. (**b**) Measurement of melanin secretion by melanin secretion assay in B16F10 cells treated with α-MSH and loliolide. (**c**) Measurement of melanin production by melanin content assay in B16F10 cells after treatment with α-MSH and loliolide. (**d**) Western blot analysis of the expression of MC1R signaling pathway proteins in α-MSH- and/or loliolide-treated B16F10 cells. Statistical significance (**b**,**c**) was evaluated using the Kruskal–Wallis/Mann–Whitney test. ^##^ *p* < 0.05 compared with normal group and ** *p* < 0.01 compared with control.

To examine the cytotoxicity of loliolide to B16F10 cells, we measured cell viability using the MTT assay. Loliolide did not show cytotoxicity to B16F10 cells up to a concentration of 100 μM (Figure 3a). α-MSH treatment increases melanin content and secretion of B16F10 cells [6]; therefore, we investigated the anti-melanogenic effect of loliolide using melanin secretion and content assays of B16F10 cells treated with loliolide and α-MSH [23,24] with arbutin as a positive control compound [30]. Loliolide effectively decreased the melanin content and secretion of B16F10 cells treated with α-MSH (Figure 3b,c). Next, we investigated the mechanism of the inhibition of melanogenesis in B16F10 cells by loliolide. α-MSH has been reported to act as a signaling molecule that can stimulate melanogenesis by activating the MC1R signaling pathway in B16F10 cells. Thus, we examined the expression of proteins in the MC1R signaling pathway by Western blot analysis to assess their role in melanogenesis and melanin secretion [31]. Loliolide effectively decreased expression of p-CREB, MITF, and tyrosinase in the MC1R signaling pathway, which were induced by α-MSH treatment (Figure 3d). This result suggests that loliolide can be an effective inhibitor of melanogenesis by decreasing the expression of key proteins in the MC1R signaling pathway.

### 2.3. Antioxidant and Anti-Melanogenesis Effect of Pj-EE

**Figure 4:**
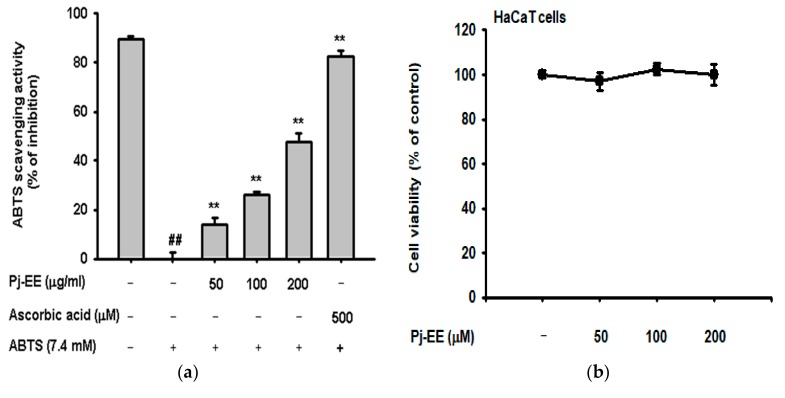

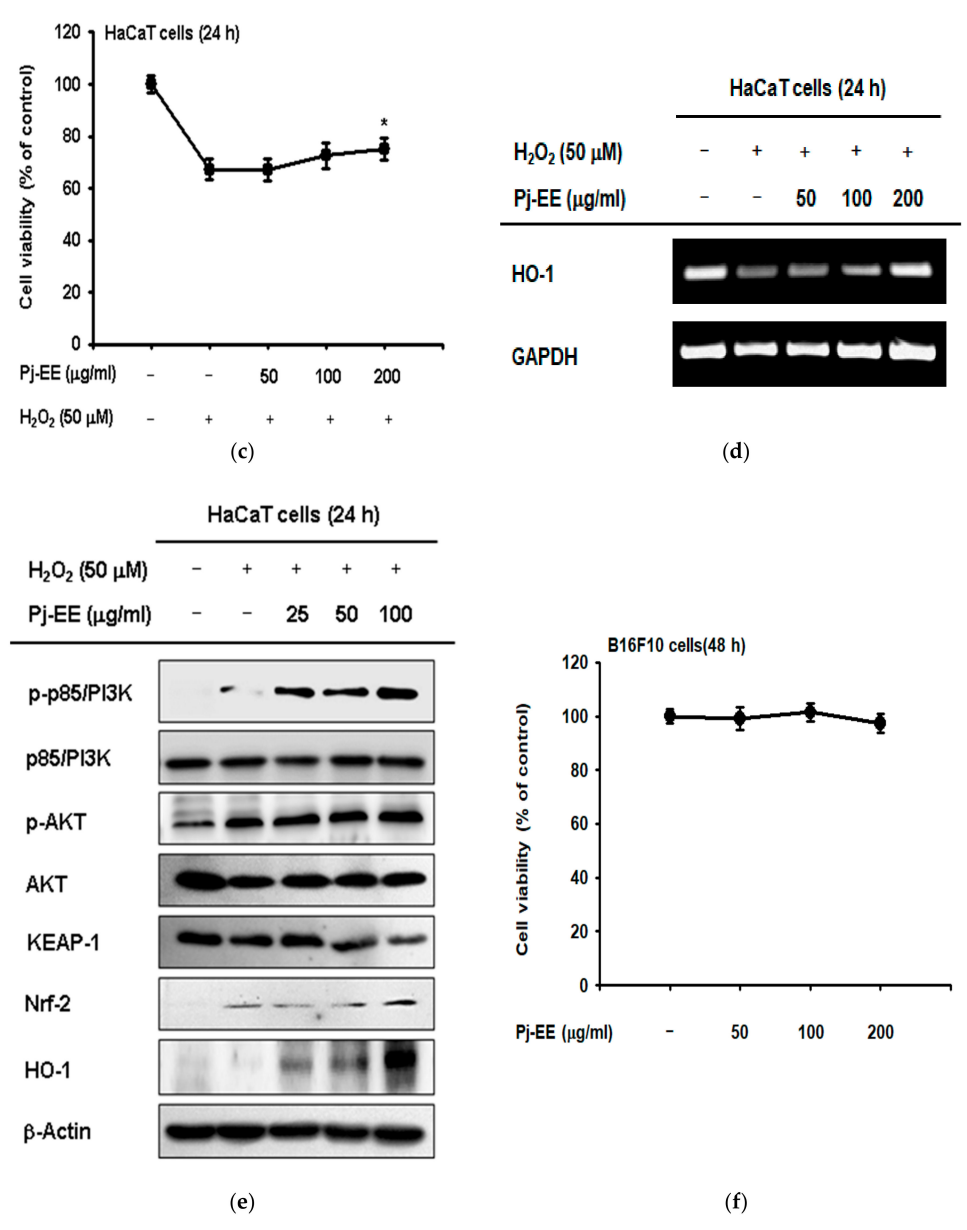

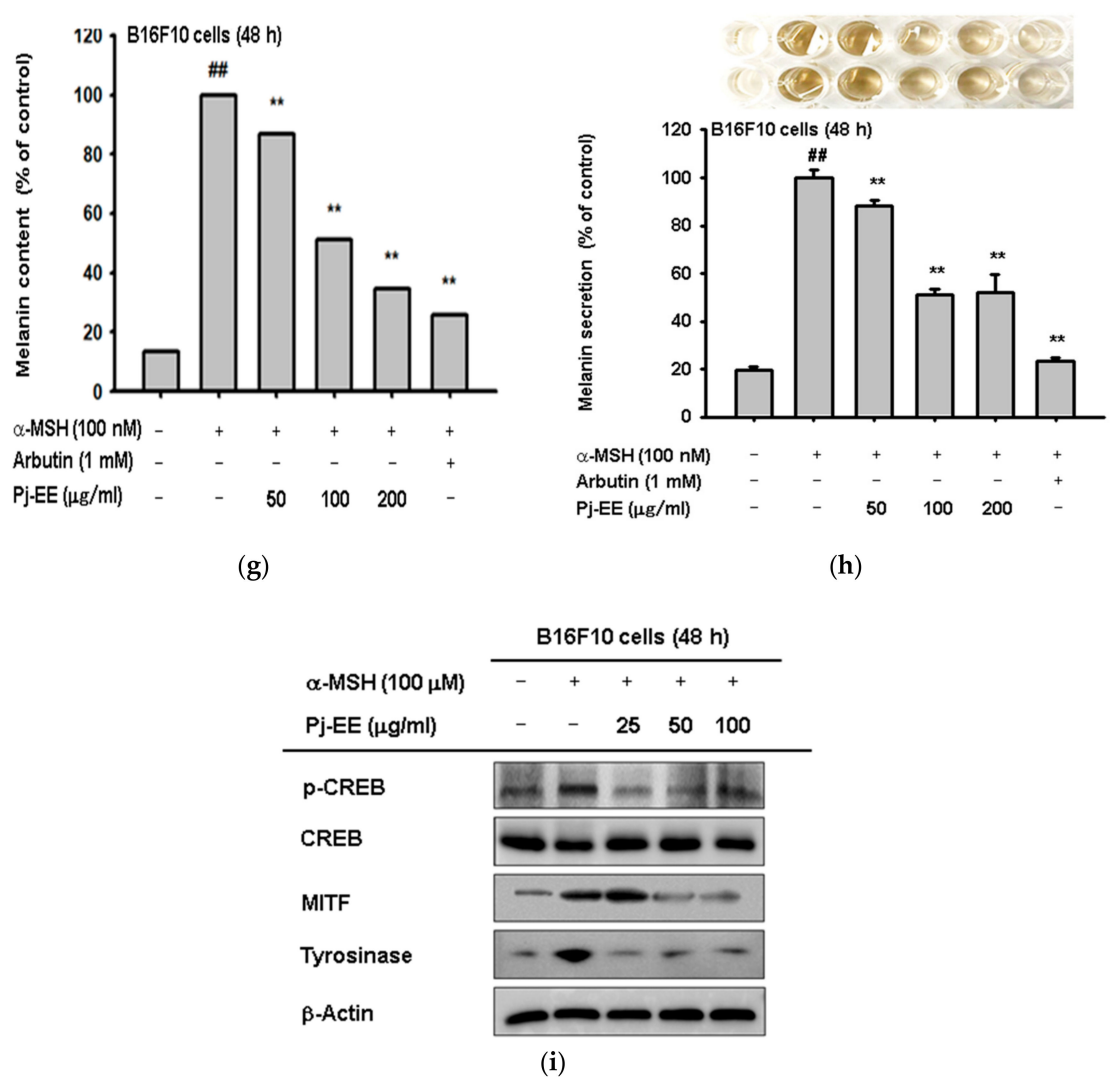
Oxidative stress-protective and anti-melanogenic effects of Pj-EE. (**a**) Measurement of the ABTS radical scavenging ability of Pj-EE. (**b**,**c**) MTT assay of the viability of HaCaT cells treated with Pj-EE alone or with Pj-EE plus H_2_O_2_. (**d**) RT-PCR analysis of *HO-1* gene expression in HaCaT cells treated with H_2_O_2_ and Pj-EE. (**e**) Western blot analysis of expression of NRF2-KEAP1 signaling pathway proteins in H_2_O_2_- and/or Pj-EE-treated HaCaT cells. (**f**) MTT assay of the viability of B16F10 cells treated with Pj-EE. (**g**) Measurement of melanin secretion by melanin secretion assay in B16F10 cells treated with α-MSH and Pj-EE. (**h**) Measurement of melanin production by melanin content assay in B16F10 cells after treatment with α-MSH and Pj-EE. (**i**) Western blot analysis of expression of MC1R signaling pathway proteins in α-MSH- and/or Pj-EE-treated B16F10 cells. Statistical significance (**a**,**c**,**g**,**h**) was evaluated using the Kruskal–Wallis/Mann–Whitney test. ^##^ *p* < 0.05 compared with normal group, and * *p* < 0.05 and ** *p* < 0.01 compared with control.

We examined the antioxidant effect of Pj-EE, known to have loliolide as major active principle [15], in this extract, using the ABTS assay with ascorbic acid as a positive control compound. Pj-EE reduced ABTS radicals with an IC_50_ value of 241.17 ± 9.32 μg/mL in the ABTS assay (Figure 4a). Cell viability was measured by MTT assay to examine the cytotoxicity of Pj-EE to B16F10 cells. Pj-EE did not show cytotoxicity to B16F10 cells up to a concentration of 200 μg/mL (Figure 4b). The viability of HaCaT cells treated with H_2_O_2_ and Pj-EE was determined by MTT assay. Pj-EE slightly inhibited the cell death caused by H_2_O_2_ (Figure 4c). To determine the effect of Pj-EE on the expression of *HO-1* in HaCaT cells, we performed RT-PCR analysis of HaCaT cells after treatment with Pj-EE, and H_2_O_2_. *HO-1* expression was significantly suppressed by H_2_O_2_, but the suppression was alleviated by Pj-EE treatment in a dose-dependent manner (Figure 4d). To measure the expression of the HO-1 protein [32], HaCaT cells were treated with H_2_O_2_ and Pj-EE together, followed by Western blot analysis. Because HO-1 is activated by the NRF2-KEAP1 signaling pathway [33], we investigated the expression of proteins in the NRF2-KEAP1 signaling pathway. Pj-EE treatment increased the expression of the PI3K, AKT, and NRF2 proteins and HO-1 (Figure 4e) [8]. These results showed that the oxidative stress-protective effect of Pj-EE was very similar to that of loliolide.

Next, we used B16F10 cells to identify Pj-EE anti-melanogenic effects. MTT assays were performed to assess Pj-EE cytotoxicity to B16F10 cells. Like loliolide, Pj-EE showed no cytotoxicity to B16F10 cells up to a concentration of 200 μg/mL (Figure 4f). To investigate the anti-melanogenic effect of Pj-EE, B16F10 cells were treated with the melanogenesis inducer α-MSH and Pj-EE and analyzed for melanin content and secretion. Arbutin was used as a positive control compound. Pj-EE effectively suppressed melanin production and secretion, both of which were increased by α-MSH (Figure 4g,h). Additionally, we investigated the molecular mechanism of the anti-melanogenic effect of Pj-EE on B16F10 cells. The important signaling pathway for melanin formation in B16F10 cells is the MC1R signaling pathway, and the MC1R signaling pathway is activated by the melanin inducer α-MSH. Thus, we analyzed the expression of proteins in the MC1R signaling pathway by Western blot. The levels of p-CREB, MITF, and tyrosinase proteins in the MC1R signaling pathway were markedly reduced in a dose-dependent manner by treatment with Pj-EE (Figure 4i) [9].

## 3. Discussion

Scientific interest in antioxidants has grown rapidly and, due to an increasingly aged population, has expanded from analysis of their dietary roles to include the development of new drugs [34]. Whereas previous studies on antioxidants focused mainly on diet to improve health and delay aging of the skin, scientists are now addressing the mechanism(s) associated with the functions of antioxidants in all organs and tissues of the human body. Our study examined the antioxidant function of loliolide, a major active component found in fresh water green algae, *Prasiola japonica*, in keratinocyte (HaCaT) cells as well as the anti-melanogenic effects on mouse melanoma (B16F10) cells. Furthermore, we investigated the molecular mechanisms of loliolide in parallel with those of Pj-EE.

Our evaluation of the cytotoxicity of loliolide and Pj-EE showed that neither exhibited cytotoxicity to HaCaT cells (Figure 2b and Figure 4b). Cell death caused by oxidative stress from H_2_O_2_ was prevented in HaCaT cells by loliolide or Pj-EE (Figure 2c and Figure 4c) and indeed, direct scavenging activity of loliolide and Pj-EE was confirmed by ABTS assay in which hydroxyl radical-generating ABTS^.^ was revealed to be converted by loliolide and Pj-EE into colorless form of ABTS^2−^ [35]. *HO-1* gene expression plays an important role in antioxidant effects. Loliolide and Pj-EE increased the expression of *HO-1* without cytotoxicity in HaCaT cells (Figure 2g and Figure 4d). *HO-1* expression is known to be regulated by the NRF2-KEAP1 signaling pathway [32]. Based on this, we examined the expression patterns of the proteins in the NRF2-KEAP1 signaling pathway by Western blot analysis. Loliolide and Pj-EE increased expression of PI3K, phosphorylated AKT (p-AKT), and NRF2 in the NRF2-KEAP1 signaling pathway (Figure 2h and Figure 4e). The Nrf2-KEAP1 proteins associate together under normal conditions, but oxidative stress induces dissociation of these proteins allowing nuclear translocation of NRF2, which can then bind to the AREs in the *HO-1* promoter to express *HO-1* gene [36]. Our findings indicate that activation of the NRF2-KEAP1 signaling pathway may increase HO-1 protein expression to enhance the antioxidant effects of loliolide and Pj-EE in skin [8]. In fact, it is well-known that HO-1 produces endogenous antioxidant bilirubin and, therefore, this molecule, as well as treated loliolide and Pj-EE, could participate in suppressing or blocking cellular risks of oxidative stress generated by H_2_O_2_ in HaCaT cells.

The amount and strength of environmental UV light is increasing due to destruction of the ozone layer by global warming [37], and this may cause increased pigmentation of the skin as well as diseases, such as skin cancer [38]. Therefore, it is necessary to study the mechanisms by which melanogenesis can be inhibited or eliminated. Therefore, we investigated whether loliolide and Pj-EE could have anti-melanogenic effects in B16F10 cells. We first showed that loliolide and Pj-EE were not cytotoxic to B16F10 cells (Figure 3a and Figure 4f). Melanogenesis is increased in B16F10 cells by α-MSH [6]; therefore, we investigated whether loliolide and Pj-EE could affect the increased melanin content and secretion of α-MSH-treated B16F10 cells. Both loliolide and Pj-EE inhibited the increase in melanin content and secretion at target concentrations and did so without cytotoxicity in B16F10 cells (Figure 3a–c and Figure 4f–h). We also studied the mechanism underpinning the anti-melanogenic effects of loliolide and Pj-EE in α-MSH-treated B16F10 cells. The major signaling pathway for melanin production is the MC1R signaling pathway [39]; thus, we analyzed the expression of MC1R signaling pathway proteins by Western blot. Loliolide and Pj-EE decreased the phosphorylation of CREB protein in the MC1R signaling pathway as well as the expression of MITF and tyrosinase proteins in B16F10 cells (Figure 3d and Figure 4i). Although testing the phosphorylation level of MITF is important to check its involvement in this process [40], the fact that p-CREB was suppressed by loliolide and Pj-EE led us to consider that controlling expression of MITF by CREB activity (Figure 3d and Figure 4i) is major inhibitory mode of action. This result raised the possibility that loliolide and Pj-EE may modulate the MC1R signaling pathway for the activation of CREB to mediate anti-melanogenic effects. 

**Figure 5:**
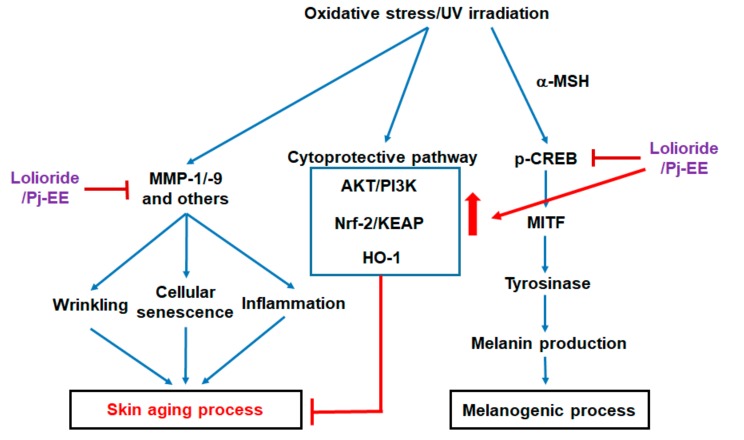
Mechanisms of oxidative stress-protective and anti-melanogenic effects of loliolide and Pj-EE. Red arrow: activation pathway induced by loliolide and Pj-EE, and blue arrow: positive pathway triggered by oxidative stress to activate downstream event.

Our skin is often exposed to oxidative stress and UV light. These stimuli can disrupt the homeostasis of the skin and can cause diseases, such as the skin cancer, pigmentation, and psoriasis [41], as also summarized in Figure 5. For this reason, maintaining homeostasis is important for preserving skin health. Antioxidants, in particular, are essential for homeostasis [42]. Therefore, antioxidants that reduce oxidative stress are needed to protect the skin from external stimuli and maintain healthy skin [43]. We demonstrated that loliolide and Pj-EE could be useful as skin antioxidants, via enhancement of HO-1 through the activation of AKT/PI3K pathway as indicated in Figure 5, as well as agents potentially protecting against skin cancer. Since there was no skin irritation under loliolide treatment conditions in our preliminary clinical study with three people (data not shown), additional research will be followed to prove these effects of loliolide and Pj-EE on human skin or artificial human skin as well as clinical study.

## 4. Materials and Methods

### 4.1. Materials

Loliolide (purity: 98% by HPLC) was purchased from Chemfaces (Wuhan, China). HaCaT and B16F10 cell lines were purchased from the American Type Culture Collection (Rockville, MD, USA). Dulbecco’s modified Eagle’s medium (DMEM), fetal bovine serum (FBS), phosphate-buffered saline (PBS), and penicillin-streptomycin were purchased from HyClone (Logan, UT, USA). 3-(4-5-Dimethylthiazol-2-yl)-2,5-diphenyltetrazolium bromide (MTT) was purchased from Amresco (Brisbane, Australia). 2,2’-Azino-bis (3-ethylbenzothiazoline-6-sulphonic acid) diammonium salt (ABTS), ascorbic acid, α-melanocyte stimulating hormone (α-MSH), TRIzol, and arbutin were purchased from Sigma Aldrich Chemical Co. (St. Louis, MO, USA). The cDNA synthesis kit was purchased from Thermo Fisher Scientific (Waltham, MA, USA). Forward and reverse primers for polymerase chain reaction (PCR) and real-time PCR were synthesized by Macrogen (Seoul, Korea), and PCR premix was purchased from Bio-D Inc. (Seoul, Korea). Polyvinylidene difluoride (PVDF) membrane was purchased from Merck Millipore (Billerica, MA, USA). Antibodies against PI3K, p-PI3K, AKT, p-AKT, Keap1, and β-actin were purchased from Cell Signaling Technology (Beverly, MA, USA). Antibodies against NRF2, HO-1, CREB, p-CREB, MITF, and tyrosinase were purchased from Santa Cruz Biotechnology (Santa Cruz, CA, USA).

### 4.2. Cell Culture and Drug Treatment

Human keratinocyte HaCaT cells and mouse melanoma B16F10 cells were cultured in DMEM supplemented with 10% fetal bovine serum and 1% penicillin-streptomycin in a 5% CO_2_ incubator at 37 °C. Loliolide and Pj-EE were dissolved in 100% dimethylsulfoxide (DMSO) and then further diluted with culture medium for preparing indicated concentrations. Equal amount of DMSO was always prepared in corresponding normal or control group as a vehicle control.

### 4.3. ABTS Assay

ABTS (7.4 mM) and potassium sulfate (2.4 mM) solutions were mixed at a ratio of 1:1 and incubated overnight at room temperature to produce ABTS radical cation. Loliolide (0–100 μM), Pj-EE (0–200 μg/mL), or ascorbic acid (500 mM) were added to a 96-well plate, and ABTS solution was added at a ratio of 1:1. The mixture was incubated at 37 °C for 30 min. Absorbance at 730 nm was measured. The ABTS scavenging activity is expressed as a percentage [44]:

ABTS scavenging activity (%) = [(A − B)/B] × 100,

where A is the absorbance of ABT, and B is the absorbance of the samples.

### 4.4. MTT Assay

Cell viability was measured using an MTT assay. HaCaT cells were plated in a 96-well plate at 3 × 10⁴ cells per well, cultured for 24 h, and then treated with loliolide (0–100 μM) or Pj-EE (0–200 μg/mL) for 24 h. B16F10 cells were plated in a 96-well plate at 5 × 10⁴ cells per well, cultured for 24 h, and treated with loliolide (0–100 μM) or Pj-EE (0–200 μg/mL) for 48 h. Cells were incubated with MTT (10 μL per well) solution for 3 h, and MTT stop solution (100 μL per well, 10% sodium dodecyl sulfate containing 1 M HCl) was added. After 8 h, the amount of solubilized formazan was determined by absorbance at 570 nm using an optical density reader (BioTek, Winooski, VT, USA) [45].

### 4.5. Extraction of Pj-EE

*Prasiola japonica* used in the experiment was supplied by the *Prasiola japonica* Research Center in Samcheok City, Gangwon-do, Korea. Samples were cut into 2 × 2-cm pieces and then extracted with 70% ethanol at room temperature for 24 h. Samples and solvents were extracted at a ratio of 1:20 (*w*/*v*). After completion of the extraction, the filtrate was filtered through 110-nm filter paper (No. 2, Advantec, Toyo Co., Tokyo, Japan), and the filtrate was concentrated using a vacuum concentrator (Eyela New Rotary Vacuum Evaporator, Rikakikai Co., Tokyo, Japan). The concentrated samples were dried using a vacuum freeze dryer (Eyela FD1, Rikakikai Co.), and the yield of the dried samples was measured. The final weight of the extract was 2.752 g (original sample: 44.87 g) with a yield of 6.13%. The dried samples were stored in a −20 °C freezer until use [46].

### 4.6. RT-PCR and Real-Time-PCR Assay

Analysis of gene expression in HaCaT and B16F10 cells was performed in six-well plates. HaCaT cells were seeded at 6 × 10^5^ cells per mL in each well and incubated for 24 h at 37 °C in a 5% CO_2_ incubator. Then, H_2_O_2_ (50 μM), loliolide (0–100 μM), and Pj-EE (0–200 μg/mL) were added, and the plates were incubated for 24 h. B16F10 cells were seeded at 8 × 10^5^ cells per mL in a six-well plate and incubated for 24 h at 37 °C in a 5% CO_2_ incubator. Then, α-MSH (100 nM), loliolide (0–100 μM), and Pj-EE (0–200 μg/mL) were added, and the plates were incubated for 48 h. TRIzol reagent was used to extract mRNA according to the manufacturer’s instructions. The concentration of the extracted mRNA was measured using a spectrophotometer, and the mRNA was used to synthesize complementary DNA (cDNA). cDNA synthesis was performed using a cDNA synthesis kit [47,48]. Gene-specific primers were designed, as reported previously [49]. RT-PCR and real-time PCR were performed using specific forward and reverse primers shown in Table 1.

### 4.7. Western Blotting Analysis

HaCaT cells were seeded in a six-well plate (5 × 10^5^ cells/mL) and cultured in a 5% CO_2_ incubator at 37 °C for 24 h. Cells were treated with loliolide (0–100 μM) or Pj-EE (0–200 μg/mL) and incubated for 24 h at 37 °C in a 5% CO_2_ incubator. B16F10 cells were seeded in a six-well plate (3 × 10^5^ cells/mL) and cultured in a 5% CO_2_ incubator at 37 °C for 24 h. Then, α-MSH, loliolide, and Pj-EE were added and incubated for 48 h at 37 °C in a 5% CO_2_ incubator. The cells were then washed three times with cold PBS and lysed with lysis buffer (20 mM Tris-HCl, pH 7.4, 2 mM EDTA, 2 mM ethylene glycoltetraacetic acid, 50 mM β-glycerophosphate, 1 mM orthovanadate, 1 mM dithiothreitol, 1% Triton X-100, 10% glycerol, 10 μg/mL aprotinin, 10 μg/mL pepstatin, 1 mM benzamidine, and 2 mM PMSF). The lysates were centrifuged at 12,000 rpm for 8 min, and the supernatant was transferred to another tube. Protein quantification was performed by Bradford analysis of the supernatant [50,51]. Cell lysates (supernatant) quantified by Bradford analysis were analyzed by Western blot. Phosphorylation or the total levels of PI3K, p-PI3K, AKT, p-AKT, Keap-1, Nrf2, HO-1, and β-actin in HaCaT cells, and phosphorylation or the total levels of CREB and p-CREB, MITF, tyrosinase, and β-actin in B16F10 cells were visualized as described previously [51,52]. All Western blot data in this study are a representative of two experiments showing similar pattern.

### 4.8. Melanin Contents and Secretion Analysis

B16F10 cells (3 × 10^5^ cells/mL) were seeded in a six-well plate and cultured in a 5% CO_2_ incubator at 37 °C for 24 h. Cells were treated with α-MSH (100 nM), loliolide (0–100 μM), Pj-EE (0–200 μg/mL), or 1 mM arbutin (positive control) and cultured in an incubator at 37 °C and 5% CO_2_ for 48 h. To measure melanin secretion, the absorbance of the cell culture medium was measured at 475 nm using a spectrophotometer [53]. To measure the melanin content, cells were dissolved in cell lysis buffer (50 mM Tris-HCl pH 7.5, 20 mM NaF, 25 mM β-glycerolphosphate pH 7.5, 120 mM NaCl, and 2% NP-40 in distilled water) and centrifuged at 12,000 rpm and 4 °C. Then, the supernatant was discarded, and the precipitate was dissolved in 1 M NaOH in 10% DMSO solution for 1 h at 60 °C. Lysate absorbance was measured at 405 nm using a spectrophotometer [24].

### 4.9. Preliminary Human Skin Irritation Patch Test

Skin toxicity of human was performed by preliminary skin irritation test with three volunteers, according to the regulation of Ministry of Food and Drug Safety, Korea. Briefly, loliolide (100 μM) applied on patch was treated every day for seven days, and from day 5, photos were taken by a digital camera. The study was approved (IRB certification No.: 1-220777-A-N-02-DICN18042, and approved data: 20 April 2018) by the Institutional Review Board Committee of Dermapro Ltd. (Seoul, Korea) and written informed consent was obtained from each volunteer.

### 4.10. Statistical Analysis

The results were analyzed using either ANOVA/Scheffe’s post hoc test or the Kruskal-Wallis/Mann–Whitney test. A value <0.05 was considered statistically significant. All statistical tests were performed using the computer program SPSS (SPSS Inc., Chicago, IL, USA).

## Figures and Tables

**Table 1:** Primers list.

Name		Sequence (5′ to 3′)
*MMP-1*	F	TGTGGTGTCTCACAGCTTCC
	R	TTGTCCCGATGATCTCCCCT
*MMP-2*	F	AAAACGGACAAAGAGTTGGCA
	R	CTGGGGCAGTCCAAAGAACT
*MMP-3*	F	TGTTAGGAGAAAGGACAGTGGTC
	R	CGTCACCTCCAATCCAAGGAA
*MMP-9*	F	ACGATGACGAGTTGTGGTCC
	R	TCGCTGGTACAGGTCGAGTA
*HO-1*	F	ACTTCCCAGAAGAGCTGCAC
	R	GCTTGAACTTGGTGGCACTG
*GAPDH*	F	CACCATCTTCCAGGAGCGAG
	R	CTCAGTGTAGCCCAGGATGC

## Abbreviations

Pj-EE	*P. japonica* ethanol extract
MMPs	Matrix metalloproteinases
NRF2	Nuclear factor (erythroid-derived 2)-like 2
HO-1	Heme oxygenase-1
MITF	Microphthalmia-associated transcription factor
α-MSH	α-melanocyte-stimulating hormone
ROS	Reactive oxygen species
UV	Ultraviolet
PI3K	Phosphatidylinositol-4,5-bisphosphate 3-kinase
AKT	Protein kinase B
MC1R	Melanocortin 1 receptor
CREB	cAMP response element-binding protein
RT-PCR	Reverse transcription-polymerase chain reaction
MTT	3-(4,5-dimethylthiazol-2-yl)-2,5-diphenyltetrazolium bromide
ABTS	2,2′-Azino-bis (3-ethylbenzothiazoline-6-sulphonic acid) diammonium salt
